# Assessing Fear and Anxiety of Corona Virus Among Dental Practitioners

**DOI:** 10.1017/dmp.2020.350

**Published:** 2020-09-11

**Authors:** V.B.P. Suryakumari, Y. Pallavi Reddy, Sarjeev Singh Yadav, Dolar Doshi, V. Surekha Reddy

**Affiliations:** Department of Conservative Dentistry & Endodontics, Government Dental College & Hospital, Hyderabad, India; Department of Public Health Dentistry, Government Dental College & Hospital, Hyderabad, India; Department of Oral Pathology, MNR Dental College & Hospital, Sangareddy, Telangana, India

**Keywords:** anxiety, COVID-19, dental practitioners, fear, India, pandemic

## Abstract

**Objective:**

Originating in China in December 2019, coronavirus disease 2019 (COVID-19) rapidly spread to more than 216 countries in the world by May 2020. Because dentists are at a higher risk of contracting the disease, the present study assessed the fear and anxiety among dental practitioners of becoming infected with COVID-19.

**Methods:**

An online cross-sectional questionnaire survey comprising of 9 questions was conducted among dental practitioners of Telangana. Age, gender, qualification, type of practice, years of practice, and place of residence were the demographic variables recorded. The response to each question was recorded in a YES or NO format, and mean fear score was calculated to categorize answers into low and high levels of fear. Comparison of mean fear score was done using t-test for 2 variables and analysis of variance for 3 or more than 3 variables. Multiple logistic regression analysis of the levels of fear with demographic variables was done. *P* < 0.05 was considered statistically significant.

**Results:**

The mean fear and anxiety score of this study population reported was high 6.57 ± 2.07, with 58.31% of the population presenting with a low level of fear and anxiety. Only qualification (*P* = 0.045) and gender (*P* = 0.035) revealed a significant difference in fear to Q7and Q8, respectively. Irrespective of the age, gender, qualification, type of practice, and years in practice, the levels of fear reported in the present study were very similar. Respondents between 41 and 60 y of age (6.70 ± 2.01 y) and those with individual practices (6.70 ± 2.06 y) exhibited high fear scores.

**Conclusions:**

The present study demonstrates cross-sectional data of fear and anxiety among dental practitioners during the COVID-19 outbreak. Heightened levels of fear observed in this study call for a nationwide analysis of fear among dentists and deliberate management strategies for the same.

Originating in China in December 2019, coronavirus disease 2019 (COVID- 19) rapidly spread to more than 216 countries in the world by May 2020.^1^ The common symptoms of severe acute respiratory syndrome coronavirus-2 (SARS-CoV2) infection include fever, cough, sore throat, myalgia, dyspnea, nausea, vomiting, diarrhea.^2^ Infected individuals can be asymptomatic and potential carriers of the virus, which may account for the cascading spread of the disease.^3,4^

Self-quarantine and isolation of symptomatic patients with physical distancing of individuals (who may be asymptomatic carriers) along with hand hygiene and cough etiquette are suggested by the World Health Organization (WHO) to abort the contact transmission of virus.^5^ Most of the countries, including India, have adopted a nationwide lockdown to halt the community transmission. Anxiety and depression are reported to be the detrimental effects of the lockdown, with mental distress and fear in public caused by pervasive viral outbreaks.^6,7^

Health-care professionals especially dentists are at a higher risk of contracting the disease, as the virus spreads primarily through droplets and aerosols.^8^ The physical effects of the disease and startling reports in social, print, and electronic media may add to the panic of dentists fearing getting infected. Fear of the disease impacts psychological well-being, as well as rational discrimination and clinical decision making. Therefore, it becomes critical to determine the level of fear of COVID-19 among the cohort of dentists. Understanding this need, the present study aimed at assessing the fear and anxiety around contracting COVID-19 among dental practitioners in the state of Telangana, India.

## METHODS

An online cross-sectional survey was conducted using Google forms and circulated through WhatsApp among dental practitioners in Telangana. Participation was voluntary, with submission of the completed questionnaire signifying informed consent. Anonymity and confidentiality was maintained throughout the survey. Inclusion criteria for the study were practicing dentists in the state of Telangana with a Smartphone and WhatsApp application and those who consented for the study. The approval for the study was obtained from the review board of ethical committee.

A questionnaire by Ahmed et al. comprising 8 questions to record fear and anxiety was used. A question whether COVID-19 vaccine would bring reassurance was added. The demographic details recorded were age, gender, qualification (Bachelor of Dental Surgery [BDS] and Master of Dental Surgery [MDS]), type of practice, years of practice, and place of residence. A pilot study was conducted to determine the sample size and to ascertain any complexity in questionnaire. Sample size was determined using the formula **n = Z** ^2^ **pq/d** ^2^. Where Z = standard normal variate value (Z-value) = 2.58, P = prevalence = 62.71, q = 100-p, and d = precision (%) = 4.

The response to each question was recorded in a YES (Score one, 1) or NO (score zero, 0) format; minimum score being 0 and maximum 1 for each question. The total score was calculated by summing up scores of all questions (ranging from 0 to 9), with a maximum possible score of 9. A mean fear score was calculated and taken as a cutoff value to categorize into low and high levels of fear. A frequency distribution of responses in the form of number and percentage was calculated. Comparison of mean fear score was done using t-test for 2 variables and analysis of variance (ANOVA) for 3 or more than 3 variables. Multiple logistic regression analysis of the levels of fear with demographic variables was done. *P* < 0.05 was considered statistically significant. Data were analyzed using SPSS software.

## RESULTS

A total of 307 complete responses were received of which the majority of the dentists were between 20 and 40 y age (185; 60.26%), with MDS qualification (206; 67.10%), and had individual practices (173; 56.35%) (Table 1). The YES/NO responses to questions 1 to 9 in number and percentage is represented in Table 2, which reveals majority of responses (80%) in the form of “YES” recorded for Questions (Qs) 1, 2, 5, and 9. Table 3 highlights the comparison of “YES” responses based on the demographic variables. A variation was observed for all variables, with an exception for Q7 and Q8 with regard to qualification and gender respectively. For Q7, “Are you anxious about the cost of treatment if you get infected?”, a marked anxiety was observed in respondents with a BDS degree (*P* = 0.035). A significantly greater number of females replied “YES” (*P* = 0.045) when compared with their male counterparts to the Q8, “Do you feel afraid when you hear that people are dying because of COVID-19?”

**TABLE 1:** Demographic Profile of Respondents

Demographic Profile	No. of Respondents (N%)
Age group (y)	
20-40	185(60.26)
41-60	122(39.74)
Gender	
Male	145(47.23)
Female	162(52.77)
Qualification	
BDS	101(32.90)
MDS	206(67.10)
Type of practice	
Affiliated with hospital	101(32.90)
Consultant	33(10.75)
Individual practice	173(56.35)
Years of practice	
<=5	110(35.83)
6-10	60(19.54)
11-15	53(17.26)
>16	84(27.37)
Total	307(100.00)

**TABLE 2:** Questions and Responses of Respondents

Question No.	Question	Yes (%)	No (%)
Q1	Are you afraid of getting infected with COVID-19 from a patient and co-worker?	258(84.04)	49 (15.96)
Q2	Are you anxious when providing treatment to a patient who is coughing or suspected of being infected with COVID-19?	277(90.23)	30 (9.77)
Q3	Do you want to close your dental practice until the number of COVID-19 cases starts declining?	210(68.40)	97(31.60)
Q4	Do you feel nervous when talking to patients in close vicinity?	206(67.10)	101(32.90)
Q5	Do you have fear that you could carry the infection from your dental practice back to your family?	288(93.81)	19(6.19)
Q6	Are you afraid of getting quarantined if get infected?	176(57.33)	131(42.67)
Q7	Are you anxious about the cost of treatment if you get infected?	129(42.02)	178(57.98)
Q8	Do you feel afraid when you hear that people are dying because of COVID-19?	214(69.71)	93(30.29)
Q9	Do you feel reassured when you hear or read about the vaccine for novel coronavirus (SARS-CoV-2)	258(84.04)	49(15.96)

**TABLE 3:** Comparison of YES Responses Based on Demographic Variables

Q No.	Age Group (y)			Gender			Qualification			Type of Practice				Years of Practice				
	20-40	41-60	*P*-Value	Male	Female	*P*-Value	BDS	MDS	*P*-Value	Affiliated with hospital	Consultant	Individual	*P*-Value	<5	6-10	11-15	>15	*P*-Value
Q1	155 (83.78)	103 (84.43)	0.88	121 (83.45)	137 (84.57)	0.78	83 (82.18)	175 (84.95)	0.53	83 (82.18)	28 (84.85)	147 (84.97)	0.82	91 (82.73)	53 (88.33)	47 (88.68)	67 (79.76)	0.39
Q2	164 (88.65)	113 (92.62)	0.25	132 (91.03)	145 (89.51)	0.65	87 (86.14)	190 (92.23)	0.09	86 (85.15)	31 (93.94)	160 (92.49)	0.10	96 (87.27)	54 (90.00)	48 (90.57)	79 (94.05)	0.47
Q3	129 (69.73)	81 (66.39)	0.53	104 (71.72)	106 (65.43)	0.23	63 (62.38)	147 (71.36)	0.11	71 (70.30)	25 (75.76)	114 (65.90)	0.47	75 (68.18)	43 (71.67)	37 (69.81)	55 (65.48)	0.87
Q4	124 (67.03)	82 (67.21)	0.97	97 (66.90)	109 (67.28)	0.94	69 (68.32)	137 (66.50)	0.75	64 (63.37)	22 (66.67)	120 (69.36)	0.59	76 (69.09)	38 (63.33)	37 (69.81)	55 (65.48)	0.83
Q5	174 (94.05)	114 (93.44)	0.82	135 (93.10)	153 (94.44)	0.62	93 (92.08)	195 (94.66)	0.37	92 (91.09)	32 (96.97)	164 (94.80)	0.34	104 (94.55)	58 (96.67)	48 (90.57)	78 (92.86)	0.56
Q6	102 (55.14)	74 (60.66)	0.33	85 (58.62)	91 (56.17)	0.66	63 (62.38)	113 (54.85)	0.21	56 (55.45)	16 (48.48)	104 (60.12)	0.41	61 (55.45)	33 (55.00)	34 (64.15)	48 (57.14)	0.72
Q7	70 (37.84)	59 (48.36)	0.06	66 (45.52)	63 (38.89)	0.24	51 (50.50)	78 (37.86)	0.035*	38 (37.62)	11 (33.33)	80 (46.24)	0.21	39 (35.45)	29 (48.33)	22 (41.51)	39 (46.43)	0.30
Q8	128 (69.19)	86 (70.49)	0.80	93 (64.14)	121 (74.69)	0.045*	68 (67.33)	146 (70.87)	0.52	69 (68.32)	20 (60.61)	125 (72.25)	0.38	79 (71.82)	39 (65.00)	39 (73.58)	57 (67.86)	0.71
Q9	152 (82.16)	106 (86.89)	0.26	121 (83.45)	137 (84.57)	0.78	84 (83.17)	174 (84.47)	0.77	87 (86.14)	26 (78.79)	145 (83.82)	0.60	93 (84.55)	51 (85.00)	40 (75.47)	74 (88.10)	0.26

*P < 0.05.

Although the mean fear and anxiety scores of this study population reported was high (6.57 ± 2.07), a high percentage of the population presented with a low level of fear and anxiety (58.31%). A comparison of mean fear and anxiety scores with demographic variables by analysis of variance (ANOVA) is represented in Table 4. Multiple logistic regression analysis of the levels of fear and anxiety with demographic variables revealed none of the predicted demographic variables had a significant association with high level of fear (Table 5).

**TABLE 4:** Comparison of Demographic Variables With Mean Fear and Anxiety Scores by 1-Way ANOVA

Demographic Profile	Levels of Fear and Anxiety			Mean + SD	*P*-Value
	Low Level (%)	High Level (%)	*P*-Value		
Age group (y)					
20-40	110(59.46)	75(40.54)	0.61	6.48+ 2.11	0.34
41-60	69 (56.56)	53(43.44)		6.70+2.01	
Gender					
Male	80(55.17)	65(44.83)	0.29	6.58+ 2.20	0.92
Female	99(61.11)	63(38.89)		6.56+1.96	
Qualification					
BDS	52(51.49)	49(48.51)	0.08	6.54+ 2.39	0.89
MDS	127(61.65)	79(38.35)		6.58+ 1.91	
Type of practice					
Affiliated with hospital	63(62.38)	38(37.62)	0.14	6.40+2.17	0.44
Consultant	23(69.70)	10(30.30)		6.39+1.84	
Individual practice	93(53.76)	80(46.24)		6.70+2.06	
Years of practice					
<=5	65(59.09)	45(40.91)	0.99	6.49**+**2.13	0.96
6-10	35(58.33)	25(41.67)		6.63**+**1.88	
11-15	30(56.60)	23(43.40)		6.64**+**2.13	
>16	49(58.33)	35(41.67)		6.57**+**2.12	
Total	179(58.31)	128(41.69)		6.57**+**2.07	

* *P* < 0.05.

**TABLE 5:** Multiple Logistic Regression Analysis of Levels of Fear and Anxiety

Profile	n	High Level (%)	OR	95% CI		
				Lower	Upper	***P*** **-Value**
Age group (y)						
20-40	185	75(40.54)	0.77	0.34	1.77	0.54
41-60	122	53(43.44)	Ref.			
Gender						
Male	145	65(44.83)	Ref.			
Female	162	63(38.89)	0.84	0.52	1.35	0.47
Qualification						
BDS	101	49(48.51)	1.34	0.80	2.25	0.26
MDS	206	79(38.35)	Ref.			
Type of practice						
Affiliated with hospital	101	38(37.62)	0.78	0.45	1.34	0.36
Consultant	33	10(30.30)	0.57	0.24	1.33	0.19
Individual practice	173	80(46.24)	Ref.			
Years of practice						
<=5	110	45(40.91)	1.36	0.51	3.68	0.54
6-10	60	25(41.67)	1.40	0.53	3.67	0.49
11-15	53	23(43.40)	1.24	0.57	2.71	0.58
>16	84	35(41.67)	Ref.			

* *P* < 0.05.

## DISCUSSION

The present public health crisis due to COVID-19 has triggered intense fear, anxiety, and stress challenging the psychological well-being among various groups.^9^ With lack of sufficient literature determining fear of coronavirus among dentists, and to provide an insight into fear and anxiety toward COVID-19, the present study was undertaken. Due to lack of a standardized questionnaire, one by Ahmed et al., which was simple, comprehensive, practical, objective, and with a good validity (0.74), was used in the present study.^10^ Although an attempt was made to include all the age strata, our study did not have any respondents older than 60, which could be due to the methodology of data collection (the phone-based application WhatsApp).

Dentists account for one of the groups of health-care workers most susceptible to this disease, as established by the evidence of transmission of virus through fomites, aerosols, and droplets.^11-13^ The viral load of SARS-CoV2 in human saliva, which was proven to be very high along with transmission even by asymptomatic carriers, places dentists into high-risk group.^11,12^ The psychological well-being of dentists may be affected and may manifest as fear of getting infected from co-workers or patients with elevated levels of anxiety when providing treatment. The same is supported by the findings of our study, wherein 80% of dentists reported positively to Q1 and 2. Likewise, in a global study that assessed anxiety and fear of dentists across 30 countries, around 90% of the respondents reported fear of getting infected from patients or co-workers.^10^

The present survey was conducted from May 9, 2020, to May 11, 2020, where the number of COVID-19 positive cases were steadily increasing both at national and state level.^14,15^ This has also affected dental practices, with 60% of our surveyed population favoring closure of practices until the number of cases start declining, indirectly signifying fear and anxiety among them.

One of the easiest and most recommended methods for reducing the transmission of the virus is practicing physical distancing. However, dental practice involves close contact with patients during routine treatments. Hence, 60% of the respondents felt nervous while talking to patients in close vicinity.

Around 90% of our study population feared carrying infection back home. The ability of the virus to thrive on inanimate surfaces for hours to days with a long incubation period makes it easy for transmission and cross-infection.^16^ This could significantly contribute to infection being carried back home and a major cause of fear among dentists as reported in a similar study, wherein 92% of dentists surveyed across 30 countries reported fear of carrying infection back home.^10^

The Ministry of Health, Medical, & Family Welfare, Government of Telangana established quarantine and isolation facilities for management of suspected and infected patients. Confusion, fear, and anxiety due to isolation and quarantine has been reported in the literature.^6^ The apprehension of getting quarantined thus affects the entire family, which has been noted in the present study with more than 50% of the respondents reporting the fear of quarantine (Q6).

Although in India, the burden of COVID-19 treatment is borne by the Government, the anticipated burden of postrecovery complications could be a factor in influencing the high level of fear of respondents to Q7 in the present study. A pronounced fear of cost of treatment when infected was noted in BDS than MDS respondents (*P* = 0.035), as it could negatively affect their prime source of income.

Greater mortality rates associated with COVID-19 is 1 of the most significant factors for fear among different sections of the population, with females susceptible to greater levels of stress and anxiety.^17-19^ In our study, females had greater fear of dying due to COVID-19 compared to male counterparts (*P* = 0.045), which could be attributed to the traditional family system still followed in India, with females as the primary care providers for households, increasing their responsibility and, thus, fear of the entire family being susceptible to consequences of COVID-19 infection. Approximately 84% of the study population had a positive attitude regarding development of a COVID-19 vaccine that could also play a vital role in controlling the pandemic.

Irrespective of the age, gender, qualification, type of practice, and years in practice, the levels of fear reported in the present study was high, similar to several studies reported in the literature.^10,17,20,21^ Overall, based on the mean fear score (6.57 ± 2.07), our study population presented a high level of fear. The augmented fear could be attributed to the probability of direct and close interaction when treating COVID-19 positive patients and a knowledge of the disease and mortality associated with the contagion. Respondents between 41 and 60 y of age exhibited high fear scores, with a mean of 6.70 ± 2.01, which depicts the fear of productive, potent, thriving practitioners, who are primarily involved in providing the necessities of life to their families. Dentists with individual practices recorded a high mean fear score (6.70 ± 2.06), which could be an effect of following the stringent guidelines and protocols issued by regulatory bodies.^22-25^ Nevertheless, those with practices affiliated with hospitals may have better support systems and facilities.

The present study, being an online survey done in a very short period of time, could not establish a cause-and-effect relationship. It being a self-reported one, the probability of social desirability bias cannot be eliminated. The results of the present study have to be carefully interpreted and cannot be generalized, as they pertain to the state of Telangana only.

## CONCLUSIONS

Pandemic outbreaks usually lead to widespread fear and mental distress in the population. The current strategies in management of COVID-19 are primarily concentrated toward controlling the disease, neglecting the psychological consequences. The present study demonstrates cross-sectional data of fear and anxiety among dental practitioners in Telangana during the COVID-19 outbreak. The heightened levels of fear observed call for a nationwide analysis of fear among dentists and deliberate management strategies for the same. The influence of gender based on the primary care provider for households and its impact on the fear of COVID-19 could form potential basis for future studies.
